# (5,5′-Dimethyl-2,2′-bipyridine-κ^2^ *N*,*N*′)diiodidomercury(II)

**DOI:** 10.1107/S160053680802953X

**Published:** 2008-09-20

**Authors:** Nasim Tadayon Pour, Amin Ebadi, Anita Abedi, Vahid Amani, Hamid Reza Khavasi

**Affiliations:** a Islamic Azad University, Shahr-e-Rey Branch, Tehran, Iran; b Department of Chemistry, Islamic Azad University, Kazeroon Branch, Kazeroon, Fars, Iran; c Department of Chemistry, Islamic Azad University, North Tehran Branch, Tehran, Iran; d Department of Chemistry, Shahid Beheshti University, Tehran 1983963113, Iran

## Abstract

In the mol­ecule of the title compound, [HgI_2_(C_12_H_12_N_2_)], the Hg^II^ atom is four-coordinated in a distorted tetra­hedral configuration by two N atoms from 5,5′-dimethyl-2,2′-bipyridine and two I atoms. There is a π–π contact between pyridine rings of adjacent molecules [centroid–centroid distance = 3.723 (5) Å].

## Related literature

For related literature, see: Ahmadi, Kalateh *et al.* (2008 ▶); Ahmadi, Khalighi *et al.* (2008 ▶); Chen *et al.* (2006 ▶); Freire *et al.* (1999 ▶); Htoon & Ladd (1976 ▶); Khalighi *et al.* (2008 ▶); Khavasi *et al.* (2008 ▶); Yousefi, Khalighi, *et al.* (2008 ▶); Yousefi, Tadayon Pour *et al.* (2008 ▶).
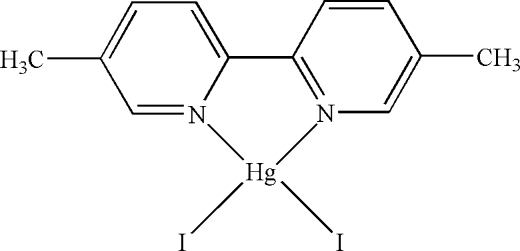


## Experimental

### 

#### Crystal data

[HgI_2_(C_12_H_12_N_2_)]
*M* *_r_* = 638.63
Orthorhombic, 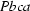

*a* = 15.0325 (8) Å
*b* = 15.0654 (8) Å
*c* = 14.0579 (10) Å
*V* = 3183.7 (3) Å^3^
*Z* = 8
Mo *K*α radiation
μ = 13.53 mm^−1^
*T* = 298 (2) K
0.35 × 0.31 × 0.20 mm

#### Data collection

Bruker SMART CCD area-detector diffractometer
Absorption correction: numerical [shape of crystal determined optically (*X-SHAPE* and *X-RED32*; Stoe & Cie (2005) ▶] *T*_min_ = 0.015, *T*_max_ = 0.075
23007 measured reflections
4306 independent reflections
3418 reflections with *I* > 2σ(*I*)
*R*_int_ = 0.083

#### Refinement

*R*[*F* ^2^ > 2σ(*F* ^2^)] = 0.055
*wR*(*F* ^2^) = 0.124
*S* = 1.19
4306 reflections
154 parameters
H-atom parameters constrained
Δρ_max_ = 1.44 e Å^−3^
Δρ_min_ = −1.51 e Å^−3^

### 

Data collection: *SMART* (Bruker, 1998 ▶); cell refinement: *SAINT* (Bruker, 1998 ▶); data reduction: *SAINT*; program(s) used to solve structure: *SHELXTL* (Sheldrick, 2008 ▶); program(s) used to refine structure: *SHELXTL*; molecular graphics: *ORTEP-3 for Windows* (Farrugia, 1997 ▶); software used to prepare material for publication: *WinGX* (Farrugia, 1999 ▶).

## Supplementary Material

Crystal structure: contains datablocks I. DOI: 10.1107/S160053680802953X/hk2531sup1.cif

Structure factors: contains datablocks I. DOI: 10.1107/S160053680802953X/hk2531Isup2.hkl

Additional supplementary materials:  crystallographic information; 3D view; checkCIF report

## Figures and Tables

**Table:** 

Hg1—I1	2.6587 (9)
Hg1—I2	2.6684 (8)
Hg1—N1	2.377 (7)
Hg1—N2	2.389 (6)

**Table:** 

I1—Hg1—I2	129.89 (3)
N1—Hg1—I1	113.59 (16)
N1—Hg1—N2	69.7 (2)
N1—Hg1—I2	106.53 (16)
N2—Hg1—I1	107.15 (15)
N2—Hg1—I2	114.22 (15)

**Fig. 1.**
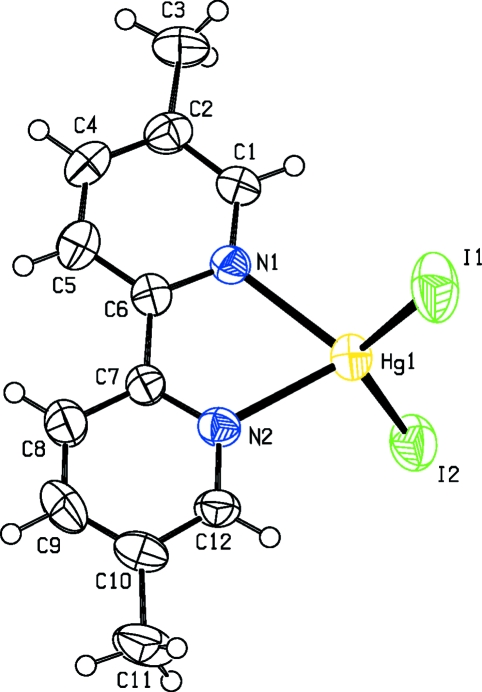
The molecular structure of the title molecule, with the atom-numbering scheme. Displacement ellipsoids are drawn at the 40% probability level.

**Fig. 2.**
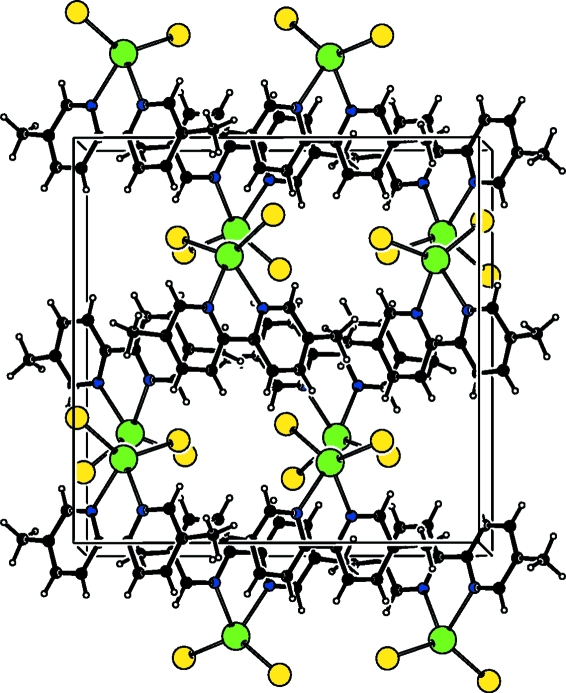
A packing diagram of the title compound.

## supplementary crystallographic information

### Comment

Recently, we reported the syntheses and crystal structures of [Zn(5,5'-dmbpy)Cl_2_], (II), (Khalighi *et al.*, 2008), [Zn(6-mbpy)Cl_2_], (III), (Ahmadi, Kalateh *et al.*, 2008), [Cd(5,5'-dmbpy)(µ-Cl)_2_]_n_, (IV), (Ahmadi, Khalighi *et al.*, 2008), [Cu(5,5'-dcbpy)(en)(H_2_O)_2_].2.5H_2_O, (V), (Yousefi, Khalighi *et al.*, 2008) and {[HgCl(dm4bt)]_2_(µ-Cl)_2_}, (VI), (Khavasi *et al.*, 2008) [where 5,5'-dmbpy is 5,5'-dimethyl-2,2'-bipyridine, 6-mbpy is 6-methyl-2,2' -bipyridine, 5,5'-dcbpy is 2,2'-bipyridine-5,5'-dicarboxylate, en is ethylene- diamine and dm4bt is 2,2'-dimethyl-4,4'-bithiazole]. There are several Hg^II^ complexes, with formula, [HgI_2_(N—N)], such as [HgI_2_(bipy)], (VII), [HgI_2_(phen)], (VIII) and [HgI_2_(2,9-dmphen)], (IX), (Freire *et al.*, 1999), [HgI_2_(bipy)][HgI_2_], (X), (Chen *et al.*, 2006), [HgI_2_(4,4'-dmbpy)], (XI), (Yousefi, Tadayon Pour *et al.*, 2008) and [HgI_2_(TMDA)], (XII), (Htoon & Ladd, 1976) [where bipy is 2,2'-bipyridine, phen is 1,10-phenanthroline, dmphen is 2,9-dimethyl-1,10-phenanthroline, 4,4'-dmbpy is 4,4'-dimethyl-2,2'-bipyridine and TMDA is tetramethylethylene- diamine] have been synthesized and characterized by single-crystal X-ray diffraction methods. We report herein the synthesis and crystal structure of the title compound, (I).

In the title compound, (Fig. 1), the Hg^II^ atom is four-coordinated in a distorted tetrahedral configuration by two N atoms from 5,5'-dimethyl-2,2'-bi- pyridine and two I atoms. The Hg—I and Hg—N bond lengths and angles (Table 1) are within normal ranges, as in (VII), (VIII) and (XI).

In the crystal structure, the π-π contact (Fig. 2) between the pyridine rings, Cg2···Cg3^i^ [symmetry code: (i) x, 1 - y, 1 - z, where Cg2 and Cg3 are centroids of the rings (N1/C1/C2/C4-C6) and (N2/C7-C10/C12), respectively] may stabilize the structure, with centroid-centroid distance of 3.723 (5) Å.

### Experimental

For the preparation of the title compound, (I), a solution of 5,5'-dimethyl -2,2'-bipyridine (0.25 g, 1.33 mmol) in methanol (10 ml) was added to a solution of HgI_2_ (0.61 g, 1.33 mmol) in methanol (5 ml) at room temperature. The suitable crystals for X-ray analysis were isolated after one week by methanol diffusion to a colorless solution in DMSO (yield; 0.62 g, 72.9%).

### Refinement

H atoms were positioned geometrically, with C-H = 0.93 and 0.96 Å for aromatic and methyl H, respectively, and constrained to ride on their parent atoms with U_iso_(H) = 1.2U_eq_(C).

### Crystal data

**Table:**

[HgI_2_(C_12_H_12_N_2_)]	*F*(000) = 2272
*M**_r_* = 638.63	*D*_x_ = 2.665 Mg m^−^^3^
Orthorhombic, *P**b**c**a*	Mo *K*α radiation, λ = 0.71073 Å
Hall symbol: -P 2ac 2ab	Cell parameters from 1768 reflections
*a* = 15.0325 (8) Å	θ = 2.4–29.3°
*b* = 15.0654 (8) Å	µ = 13.53 mm^−^^1^
*c* = 14.0579 (10) Å	*T* = 298 K
*V* = 3183.7 (3) Å^3^	Prism, colorless
*Z* = 8	0.35 × 0.31 × 0.20 mm

### Data collection

**Table:**

Bruker SMART CCD area-detector diffractometer	4306 independent reflections
Radiation source: fine-focus sealed tube	3418 reflections with *I* > 2σ(*I*)
graphite	*R*_int_ = 0.083
φ and ω scans	θ_max_ = 29.3°, θ_min_ = 2.4°
Absorption correction: numerical shape of crystal determined optically (X-SHAPE and X-RED32; Stoe& Cie, 2005)	*h* = −20→19
*T*_min_ = 0.015, *T*_max_ = 0.075	*k* = −20→17
23007 measured reflections	*l* = −19→19

### Refinement

**Table:**

Refinement on *F*^2^	Primary atom site location: structure-invariant direct methods
Least-squares matrix: full	Secondary atom site location: difference Fourier map
*R*[*F*^2^ > 2σ(*F*^2^)] = 0.055	Hydrogen site location: inferred from neighbouring sites
*wR*(*F*^2^) = 0.124	H-atom parameters constrained
*S* = 1.19	*w* = 1/[σ^2^(*F*_o_^2^) + (0.0347*P*)^2^ + 17.4498*P*] where *P* = (*F*_o_^2^ + 2*F*_c_^2^)/3
4306 reflections	(Δ/σ)_max_ = 0.010
154 parameters	Δρ_max_ = 1.44 e Å^−^^3^
0 restraints	Δρ_min_ = −1.51 e Å^−^^3^

### Special details

**Table:**

Geometry. All e.s.d.'s (except the e.s.d. in the dihedral angle between two l.s. planes) are estimated using the full covariance matrix. The cell e.s.d.'s are taken into account individually in the estimation of e.s.d.'s in distances, angles and torsion angles; correlations between e.s.d.'s in cell parameters are only used when they are defined by crystal symmetry. An approximate (isotropic) treatment of cell e.s.d.'s is used for estimating e.s.d.'s involving l.s. planes.
Refinement. Refinement of *F*^2^ against ALL reflections. The weighted *R*-factor *wR* and goodness of fit *S* are based on *F*^2^, conventional *R*-factors *R* are based on *F*, with *F* set to zero for negative *F*^2^. The threshold expression of *F*^2^ > σ(*F*^2^) is used only for calculating *R*-factors(gt) *etc*. and is not relevant to the choice of reflections for refinement. *R*-factors based on *F*^2^ are statistically about twice as large as those based on *F*, and *R*- factors based on ALL data will be even larger.

### Fractional atomic coordinates and isotropic or equivalent isotropic displacement parameters (Å^2^)

**Table:**

	*x*	*y*	*z*	*U*_iso_*/*U*_eq_	
Hg1	0.11964 (2)	0.71065 (2)	0.60202 (3)	0.05197 (12)	
I1	0.00810 (7)	0.80915 (5)	0.50004 (7)	0.0859 (3)	
I2	0.25140 (5)	0.76000 (5)	0.71769 (6)	0.0687 (2)	
N1	0.1696 (4)	0.5812 (5)	0.5214 (5)	0.0403 (14)	
N2	0.0387 (4)	0.5836 (4)	0.6554 (5)	0.0381 (13)	
C1	0.2371 (5)	0.5833 (6)	0.4598 (6)	0.0463 (18)	
H1	0.2633	0.6377	0.4460	0.056*	
C2	0.2697 (6)	0.5082 (7)	0.4155 (6)	0.049 (2)	
C3	0.3485 (8)	0.5156 (9)	0.3481 (9)	0.077 (3)	
H3A	0.3986	0.5399	0.3816	0.093*	
H3B	0.3333	0.5538	0.2959	0.093*	
H3C	0.3635	0.4578	0.3243	0.093*	
C4	0.2299 (7)	0.4279 (7)	0.4391 (8)	0.063 (3)	
H4	0.2506	0.3755	0.4121	0.076*	
C5	0.1604 (7)	0.4257 (6)	0.5019 (7)	0.053 (2)	
H5	0.1324	0.3723	0.5162	0.063*	
C6	0.1322 (5)	0.5044 (5)	0.5440 (5)	0.0387 (16)	
C7	0.0586 (5)	0.5057 (5)	0.6156 (5)	0.0378 (15)	
C8	0.0140 (6)	0.4291 (6)	0.6412 (7)	0.054 (2)	
H8	0.0270	0.3754	0.6118	0.065*	
C9	−0.0503 (6)	0.4341 (6)	0.7113 (7)	0.054 (2)	
H9	−0.0805	0.3829	0.7297	0.065*	
C10	−0.0703 (6)	0.5135 (6)	0.7542 (6)	0.0477 (19)	
C11	−0.1370 (7)	0.5206 (8)	0.8337 (8)	0.069 (3)	
H11A	−0.1831	0.5615	0.8159	0.083*	
H11B	−0.1078	0.5417	0.8901	0.083*	
H11C	−0.1625	0.4633	0.8458	0.083*	
C12	−0.0244 (5)	0.5873 (6)	0.7226 (6)	0.0465 (18)	
H12	−0.0379	0.6422	0.7492	0.056*	

### Atomic displacement parameters (Å^2^)

**Table:**

	*U*^11^	*U*^22^	*U*^33^	*U*^12^	*U*^13^	*U*^23^
Hg1	0.0544 (2)	0.04113 (17)	0.0603 (2)	0.00092 (15)	0.00422 (17)	−0.00082 (14)
I1	0.0978 (6)	0.0521 (4)	0.1078 (7)	0.0131 (4)	−0.0354 (5)	0.0050 (4)
I2	0.0660 (4)	0.0555 (4)	0.0845 (5)	0.0031 (3)	−0.0154 (4)	−0.0110 (3)
N1	0.039 (3)	0.044 (3)	0.037 (3)	0.001 (3)	0.003 (3)	−0.002 (3)
N2	0.035 (3)	0.039 (3)	0.041 (3)	0.002 (3)	0.000 (3)	0.001 (3)
C1	0.040 (4)	0.053 (5)	0.046 (4)	−0.002 (4)	0.009 (3)	−0.006 (4)
C2	0.041 (4)	0.072 (6)	0.035 (4)	0.012 (4)	0.000 (3)	−0.002 (4)
C3	0.061 (6)	0.098 (9)	0.073 (7)	0.016 (6)	0.029 (6)	−0.004 (6)
C4	0.070 (7)	0.058 (6)	0.062 (6)	0.019 (5)	0.001 (5)	−0.020 (5)
C5	0.058 (5)	0.045 (4)	0.056 (5)	0.003 (4)	0.004 (4)	−0.002 (4)
C6	0.038 (4)	0.038 (4)	0.040 (4)	0.001 (3)	−0.004 (3)	−0.002 (3)
C7	0.039 (4)	0.040 (4)	0.035 (4)	0.003 (3)	−0.009 (3)	−0.001 (3)
C8	0.057 (5)	0.047 (5)	0.058 (5)	−0.009 (4)	−0.004 (4)	−0.002 (4)
C9	0.051 (5)	0.051 (5)	0.062 (6)	−0.016 (4)	0.003 (4)	0.007 (4)
C10	0.038 (4)	0.057 (5)	0.048 (5)	−0.001 (4)	−0.005 (3)	0.010 (4)
C11	0.059 (6)	0.080 (7)	0.067 (6)	−0.001 (5)	0.031 (5)	0.000 (5)
C12	0.042 (4)	0.048 (4)	0.050 (5)	0.003 (4)	0.009 (4)	−0.003 (4)

### Geometric parameters (Å, °)

**Table:**

Hg1—I1	2.6587 (9)	C6—N1	1.325 (10)
Hg1—I2	2.6684 (8)	C6—C7	1.496 (11)
Hg1—N1	2.377 (7)	C7—N2	1.335 (10)
Hg1—N2	2.389 (6)	C7—C8	1.382 (12)
C1—N1	1.335 (10)	C8—C9	1.384 (14)
C1—C2	1.380 (13)	C8—H8	0.9300
C1—H1	0.9300	C9—C10	1.373 (13)
C2—C4	1.390 (15)	C9—H9	0.9300
C2—C3	1.521 (13)	C10—C12	1.382 (12)
C3—H3A	0.9600	C10—C11	1.505 (13)
C3—H3B	0.9600	C11—H11A	0.9600
C3—H3C	0.9600	C11—H11B	0.9600
C4—C5	1.368 (14)	C11—H11C	0.9600
C4—H4	0.9300	C12—N2	1.339 (10)
C5—C6	1.392 (11)	C12—H12	0.9300
C5—H5	0.9300		
			
I1—Hg1—I2	129.89 (3)	C4—C5—C6	119.1 (9)
N1—Hg1—I1	113.59 (16)	C4—C5—H5	120.5
N1—Hg1—N2	69.7 (2)	C6—C5—H5	120.5
N1—Hg1—I2	106.53 (16)	N1—C6—C5	120.9 (8)
N2—Hg1—I1	107.15 (15)	N1—C6—C7	117.6 (7)
N2—Hg1—I2	114.22 (15)	C5—C6—C7	121.5 (7)
C1—N1—Hg1	122.0 (6)	N2—C7—C8	121.1 (8)
C6—N1—Hg1	117.9 (5)	N2—C7—C6	117.4 (7)
C6—N1—C1	119.9 (7)	C8—C7—C6	121.5 (7)
C7—N2—Hg1	117.3 (5)	C7—C8—C9	118.7 (9)
C7—N2—C12	119.4 (7)	C7—C8—H8	120.7
C12—N2—Hg1	123.3 (5)	C9—C8—H8	120.7
N1—C1—C2	122.9 (9)	C10—C9—C8	120.8 (8)
N1—C1—H1	118.5	C10—C9—H9	119.6
C2—C1—H1	118.5	C8—C9—H9	119.6
C1—C2—C4	116.9 (8)	C9—C10—C12	116.8 (8)
C1—C2—C3	119.9 (10)	C9—C10—C11	122.3 (9)
C4—C2—C3	123.2 (9)	C12—C10—C11	120.9 (9)
C2—C3—H3A	109.5	C10—C11—H11A	109.5
C2—C3—H3B	109.5	C10—C11—H11B	109.5
H3A—C3—H3B	109.5	H11A—C11—H11B	109.5
C2—C3—H3C	109.5	C10—C11—H11C	109.5
H3A—C3—H3C	109.5	H11A—C11—H11C	109.5
H3B—C3—H3C	109.5	H11B—C11—H11C	109.5
C5—C4—C2	120.3 (9)	N2—C12—C10	123.2 (8)
C5—C4—H4	119.8	N2—C12—H12	118.4
C2—C4—H4	119.8	C10—C12—H12	118.4
			
I1—Hg1—N1—C1	82.9 (6)	C5—C6—N1—C1	−2.1 (12)
I1—Hg1—N1—C6	−101.5 (5)	C7—C6—N1—C1	178.3 (7)
I2—Hg1—N1—C1	−66.4 (6)	C5—C6—N1—Hg1	−177.8 (6)
I2—Hg1—N1—C6	109.2 (5)	C7—C6—N1—Hg1	2.6 (9)
N2—Hg1—N1—C1	−176.7 (7)	N1—C6—C7—N2	−3.3 (11)
N2—Hg1—N1—C6	−1.1 (5)	C5—C6—C7—N2	177.1 (8)
I1—Hg1—N2—C7	108.7 (5)	N1—C6—C7—C8	178.0 (8)
I1—Hg1—N2—C12	−71.7 (6)	C5—C6—C7—C8	−1.6 (12)
I2—Hg1—N2—C7	−100.3 (5)	C8—C7—N2—C12	1.4 (12)
I2—Hg1—N2—C12	79.3 (6)	C6—C7—N2—C12	−177.3 (7)
N1—Hg1—N2—C7	−0.8 (5)	C8—C7—N2—Hg1	−179.0 (6)
N1—Hg1—N2—C12	178.9 (7)	C6—C7—N2—Hg1	2.3 (9)
C2—C1—N1—C6	1.5 (13)	N2—C7—C8—C9	−1.9 (13)
C2—C1—N1—Hg1	177.0 (6)	C6—C7—C8—C9	176.7 (8)
N1—C1—C2—C4	−1.1 (13)	C7—C8—C9—C10	0.6 (15)
N1—C1—C2—C3	−178.3 (9)	C8—C9—C10—C12	1.2 (14)
C1—C2—C4—C5	1.4 (14)	C8—C9—C10—C11	−177.2 (9)
C3—C2—C4—C5	178.5 (10)	C9—C10—C12—N2	−1.8 (13)
C2—C4—C5—C6	−2.1 (15)	C11—C10—C12—N2	176.6 (9)
C4—C5—C6—N1	2.5 (14)	C10—C12—N2—C7	0.6 (13)
C4—C5—C6—C7	−178.0 (8)	C10—C12—N2—Hg1	−179.1 (6)
